# Essential element and toxic metal dyshomeostasis in childhood autism spectrum disorder: a comprehensive narrative review of correlations, tissue-specific distribution, and multifactorial regulation

**DOI:** 10.3389/fnbeh.2026.1944510

**Published:** 2026-09-23

**Authors:** Qian Du, Qunying Zhou, Li Yu, Yunchao Ye, Xiaoling Wu

**Affiliations:** 1 Meishan Women and Children’s Hospital, Alliance Hospital of West China Second University Hospital, Meishan, Sichuan, China; 2 Hongya County Psychiatric Hospital, Meishan, Sichuan, China; 3 Meishan Hospital of Traditional Chinese Medicine, Meishan, Sichuan, China; 4 Hongya County People’s Hospital, Meishan, Sichuan, China

**Keywords:** childhood autism spectrum disorder, essential elements, tissue-specific distribution, toxic metals, trace elements

## Abstract

Autism spectrum disorder (ASD) is a neurodevelopmental disorder characterized by impaired social communication and repetitive stereotyped behaviors, with a rising global prevalence. Its etiology involves genetic, environmental, and epigenetic interactions, among which essential elements [including trace elements such as zinc (Zn), selenium (Se), copper (Cu), iron (Fe), manganese (Mn) and macrominerals like magnesium (Mg)] and toxic metals (lead (Pb), mercury (Hg), cadmium (Cd)) are key environmental regulators of neurodevelopment. This narrative review systematically summarizes the association between these elements and ASD in children. Core findings confirm that Zn/Se/Fe/Mg deficiency, tissue-specific Cu imbalance (elevated plasma, reduced hair), Pb/Hg accumulation, and Zn-Cu ratio imbalance are significantly correlated with ASD occurrence and severity. These abnormalities disrupt neurotransmitter homeostasis, oxidative stress balance, and synaptic plasticity, which pathways closely linked to ASD pathogenesis. Critically, we highlight the tissue-specific distribution patterns of these elements (differences across blood, hair, urine, and nails) and the multifactorial regulatory network involving genetic variants (e.g., metal transporter genes, metallothioneins), environmental exposures (diet, pollutants, pesticides), individual differences (age, gender), and comorbidities (gastrointestinal dysfunction). Intervention evidence shows that supplementing deficient essential elements or reducing toxic metal exposure may alleviate ASD symptoms, though study quality and methodological heterogeneity limit causal inferences. This review provides a theoretical basis for ASD pathogenesis research, early diagnosis, and targeted element-based interventions.

## Graphical Abstract

**Figure:**
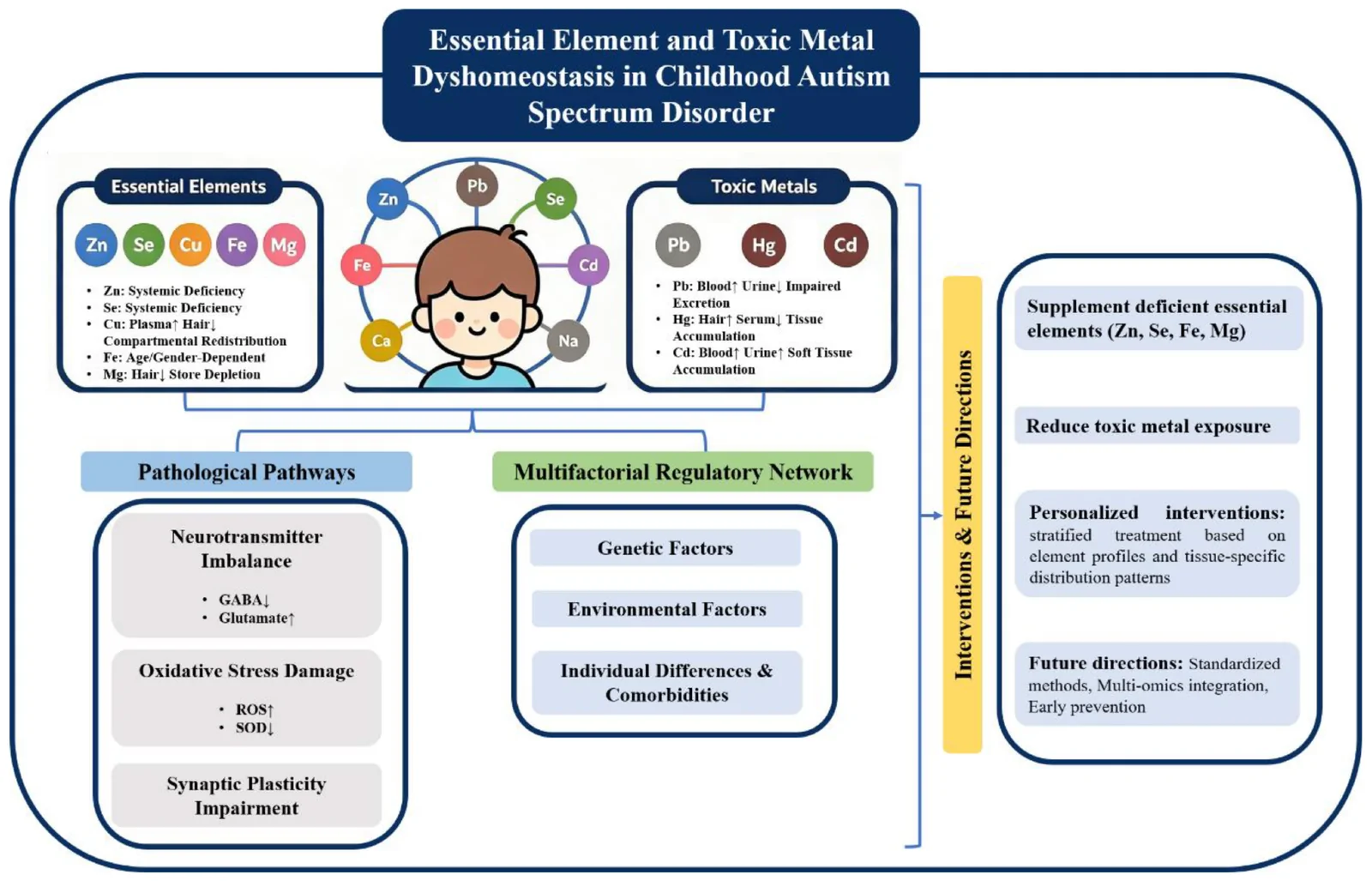
Essential element and toxic metal dyshomeostasis in childhood autism spectrum disorder: from mechanisms to interventions.

## 1 Introduction

Autism spectrum disorder (ASD) is a severe neurodevelopmental disorder that typically manifests in early childhood, with core diagnostic criteria (per DSM-5) including deficits in social interaction/communication and restricted, repetitive behaviors/interests (Awadh et al., 2023; Lord et al., 2017). The global prevalence of ASD has increased dramatically from 1/150 in 2000 to 1/36 in 2023, with a male-to-female incidence ratio of approximately 4:1, making it a major public health concern worldwide (Maenner et al., 2023; Nevison, 2014). The etiology of ASD is complex and multifactorial, involving genetic, environmental, and epigenetic interactions (Kim and Leventhal, 2015). Genetic factors contribute to ASD susceptibility, while environmental triggers include toxin exposure, dietary nutrition, and infections (Kim and Leventhal, 2015). Notably, gut-brain axis mediates the crosstalk between gut microbiota and central nervous system (CNS) function through immune, neuroendocrine, and neurotransmitter pathways, and its dysfunction is closely associated with ASD core symptoms such as social withdrawal and repetitive behaviors (Fairbrass et al., 2022; Kim and Leventhal, 2015; Varghese et al., 2017).

Trace elements are inorganic substances required by the human body in minute amounts (typically < 100 mg/day) that are critical for enzymatic activity, neurotransmitter synthesis, and antioxidant defense (Fiore et al., 2020). Essential trace elements (Zn, Se, Cu, Fe, Mn) and macrominerals (Mg, Ca) are indispensable for normal neurodevelopment, while toxic metals (Pb, Hg, Cd, As) accumulate in the body and induce neurotoxicity (Fiore et al., 2020). Abnormal levels (deficiency or excess) of these elements can disrupt neuronal function, trigger oxidative stress, and impair synaptic plasticity, which pathological processes closely associated with ASD core symptoms (social impairment, repetitive behaviors) (Bjørklund et al., 2018; Farina et al., 2013; Fiore et al., 2020; Skalny et al., 2017b). For example, Zn deficiency may disrupt GABA and glutamate metabolism, while Pb accumulation replaces essential metals in cellular enzymes, both contributing to ASD-related behaviors. However, existing research on essential elements and toxic metals and ASD remains inconsistent. Studies have reported conflicting findings on element levels in ASD children across biological samples (serum, hair, urine, nails) (Ouisselsat et al., 2025; Skalny et al., 2017b), and the specific mechanisms linking individual elements to ASD symptoms (e.g., social impairment, repetitive behaviors) remain unclear (Skalny et al., 2017b; Zhang J. et al., 2021).

Given the critical role of essential elements in neurodevelopment and their potential as modifiable targets for intervention, a comprehensive understanding of their complex relationship with ASD is urgently needed. This review emphasizes two under-explored dimensions: (1) the tissue-specific distribution patterns of elements across different biological matrices in ASD children, and (2) the multifactorial regulatory network (genetic, environmental, individual, and disease-related factors) that governs element homeostasis. This review focuses on the associations between key essential elements and toxic metals and ASD in children, clarifies the direction and nature of these associations, and discusses mediating factors.

## 2 Methods

This is a narrative review, not a systematic review. To ensure transparency and critical evaluation of the literature, we applied the following methodology:

Search Strategy: We searched PubMed, Scopus, and Web of Science for peer-reviewed articles published between January 2000 and December 2025 using the following keywords: (“autism spectrum disorder” OR “ASD” OR “autism”) AND (“trace elements” OR “zinc” OR “selenium” OR “copper” OR “iron” OR “magnesium” OR “manganese” OR “lead” OR “mercury” OR “cadmium” OR “toxic metals”) AND (“children” OR “pediatric” OR “childhood”). Additional articles were identified through reference lists of included studies and relevant reviews.

Inclusion Criteria: (1) Case-control, cohort, or cross-sectional studies measuring element concentrations in biological samples (blood, serum, plasma, hair, urine, nails) of children with ASD (age ≤ 18 years) compared to neurotypical controls; (2) intervention studies (supplementation or chelation) reporting ASD symptom outcomes; (3) studies published in English.

Exclusion Criteria: (1) Studies without a control group; (2) studies on adults only; (3) case reports or series; (4) studies not reporting quantitative element concentrations.

Quality Assessment: We evaluated the methodological quality of included studies using a customized checklist based on: (1) sample size (≥50 per group considered higher quality); (2) analytical method validation (e.g., ICP-MS with certified reference materials); (3) matching for age and gender; (4) control for confounders (diet, GI status, medication); (5) clear diagnostic criteria for ASD (DSM or ADOS). Studies meeting ≥4 criteria were considered high quality (Figure 1).

**FIGURE 1:**
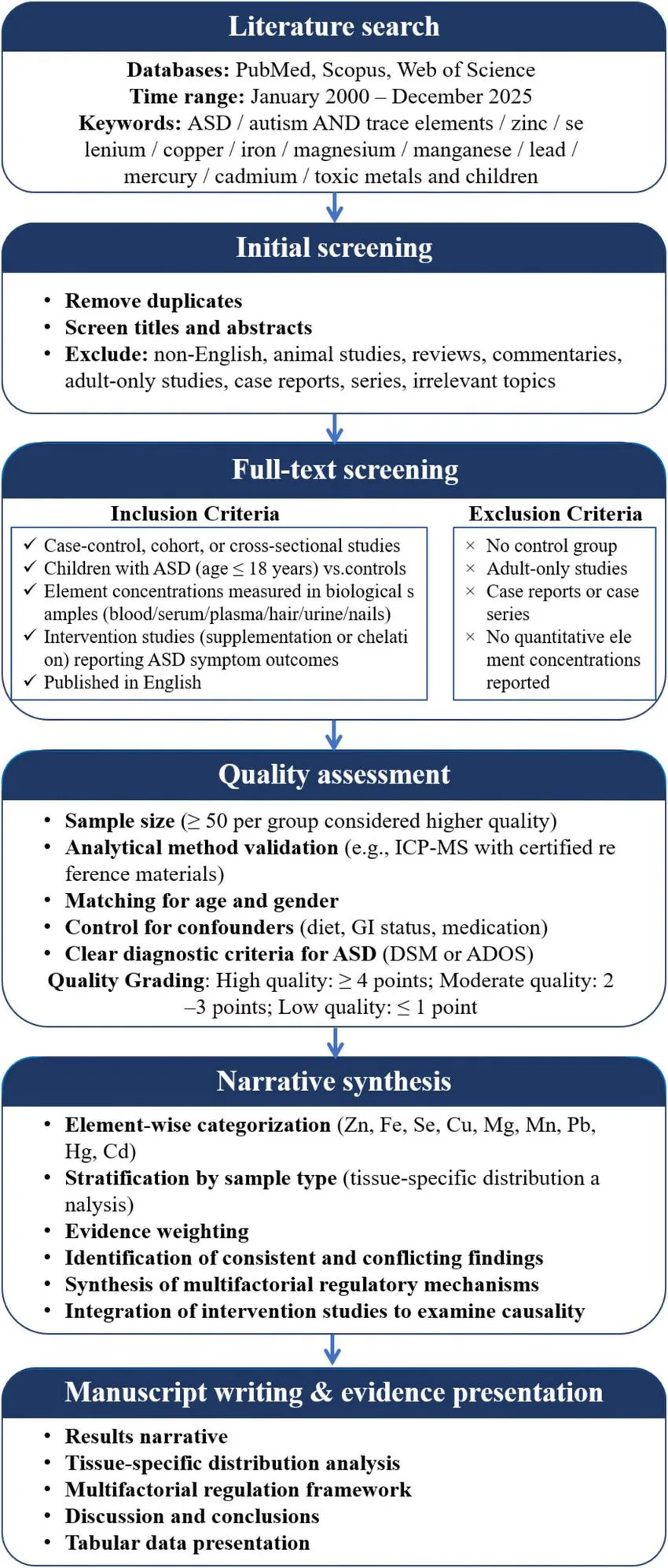
Methodological flowchart of this narrative review.

## 3 Essential elements and toxic metals in ASD children

Metabolic disorders of essential elements (including trace elements Zn, Cu, Fe, Se, Mn and macrominerals Mg, Ca) are common in children with ASD (Saghazadeh et al., 2017). Abnormal element levels induce ASD core symptoms by disrupting three key pathological pathways: neurotransmitter homeostasis, antioxidant defense system function, and synaptic plasticity regulation, which were potential changes in these levels may affect the treatment of ASD (Bjorklund, 2013; Hagmeyer et al., 2018; Yasuda et al., 2011; Figure 2).

**FIGURE 2:**
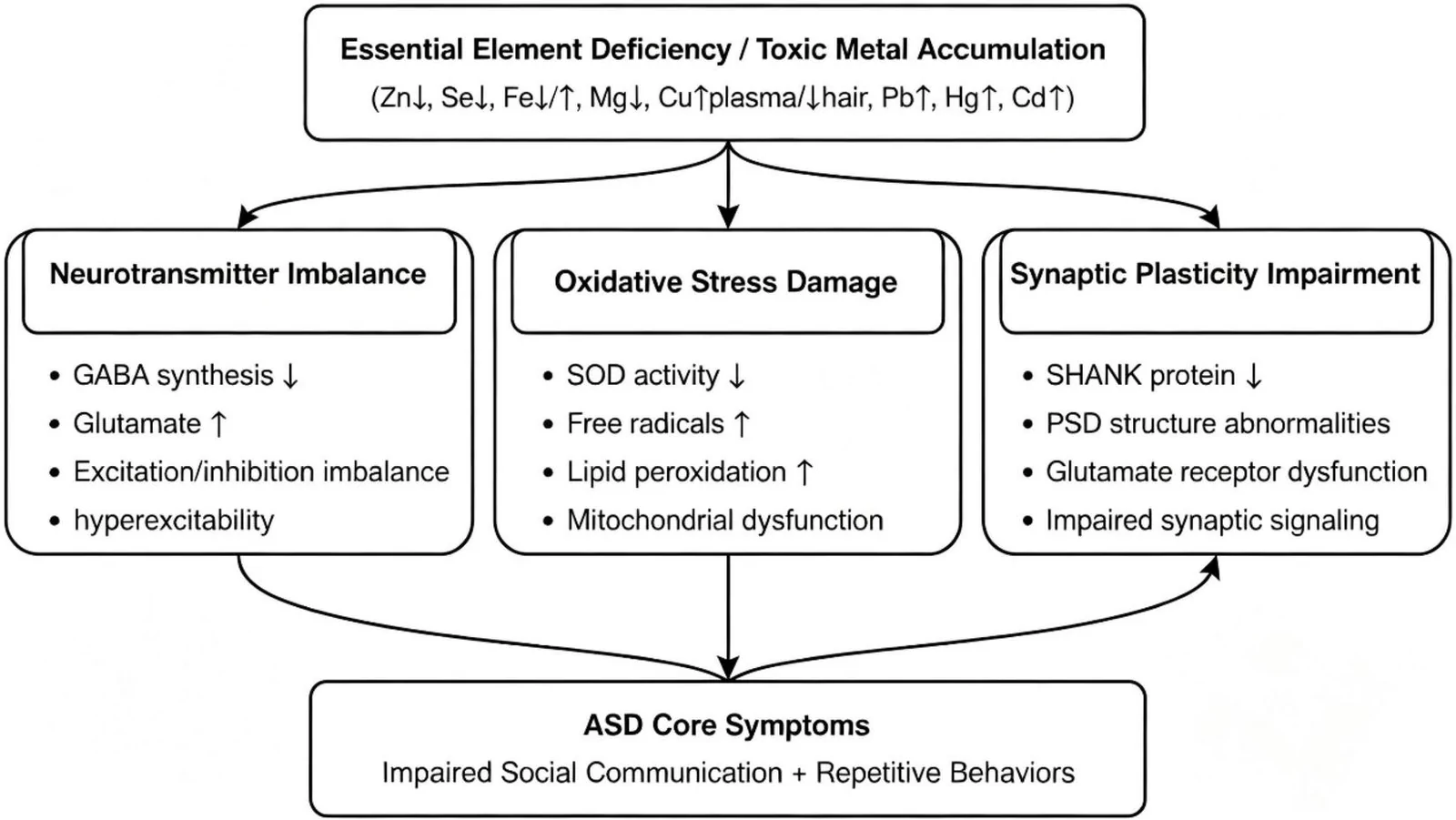
Three key pathological pathways disrupted by element dyshomeostasis in ASD.

### 3.1 Zn

Zn plays an important role in human growth and development, immune regulation, taste, and smell, etc. It is an important component of more than 200 enzymes in the human body and participates in various physiological processes such as DNA synthesis, protein metabolism, cell division, and gene expression (Livingstone, 2015). Zn deficiency may lead to the occurrence and development of autism through multiple mechanisms. From the perspective of neurotransmitters, Zn deficiency will affect the synthesis and metabolism of neurotransmitters. Zn is involved in the related metabolic processes of neurotransmitters such as gamma-aminobutyric acid (GABA) and glutamate. When there is a Zn deficiency, the synthesis of GABA decreases, and the inhibitory function of the brain weakens, making it easy to cause excessive excitation of neurons (Lin et al., 2024). At the same time, abnormal uptake and metabolism of glutamate may lead to the accumulation of glutamate in the synaptic cleft, overactivate glutamate receptors, and trigger excitotoxicity, causing damage to nerve cells (Ferraro et al., 2025). This imbalance of neurotransmitters will affect the normal transmission of nerve signals, and further lead to symptoms such as social impairment, delayed language development, and repetitive and stereotyped behaviors in children with autism (Saleh et al., 2024).

Zn also plays an important role in the antioxidant system. Zn is a component of antioxidant enzymes such as superoxide dismutase (SOD). These antioxidant enzymes can scavenge free radicals in the body and protect cells from oxidative damage (Johnson and Giulivi, 2005). When there is a Zn deficiency, the activity of antioxidant enzymes decreases, and a large number of free radicals accumulate in the body, triggering oxidative stress. Oxidative stress will damage the structure and function of the nerve cell membrane, leading to changes in the permeability of nerve cells and affecting the release of neurotransmitters and the function of receptors (Thorsen et al., 2022). Oxidative stress will also damage organelles such as mitochondria, affect the energy metabolism of cells, and seriously affect the normal function of nerve cells (Cortelazzo et al., 2016). In addition, Zn also plays an important role in the plasticity of nerve synapses. It can affect the function and expression of neurotransmitter receptors and participate in the process of nerve signal transduction. In particular, it plays a crucial role in regulating the SH3 and multiple ankyrin (SHANK) protein (a scaffold protein that regulates the formation, organization, and plasticity of excitatory synapses), which is essential for organizing excitatory synapses (Conti et al., 2024).

A number of clinical studies have pointed out that the Zn concentration in patients with ASD is significantly negatively correlated with the severity of the disease. In children with severe ASD, the Zn levels in serum, hair, and whole blood are generally reduced (Guo et al., 2020; Hawari et al., 2020; Nabgha-e-Amen et al., 2020), and Zn deficiency is often accompanied by abnormal accumulation of neurotoxic metals such as Pb, barium (Ba), and Hg (Grabrucker et al., 2014; Hawari et al., 2020; Tabatadze et al., 2015). During the critical period of neurodevelopment, Zn participates in the regulation of brain morphogenesis and the maturation of oligodendrocytes through Zn finger proteins (Al-Naama et al., 2020; Bourassa et al., 2018), and its functional disorder may induce abnormal brain structure and ASD-related neurodevelopmental disorders (Al-Naama et al., 2020; Bou Khalil, 2019). Zn deficiency can impair the functional activity of Proline-rich synapse-associated protein-1 (ProSAP1)/SH3 and multiple ankyrin repeat domains 2 (SHANK2) and Proline-rich synapse-associated protein-2 (ProSAP2)/SH3 and multiple ankyrin repeat domains 3 (SHANK3) proteins in the postsynaptic density (PSD; Grabrucker et al., 2014). Mutations in the SHANK gene (especially SHANK3) are related to ASD, and its encoded protein is crucial for the assembly of excitatory synapses (Leblond et al., 2014). It should be noted that all the above studies are cross-sectional group comparison studies, and the observed Zn deficiency in ASD children only indicates a significant correlation between Zn metabolism abnormality and ASD, and cannot directly prove the causal relationship that Zn deficiency causes ASD. In fact, Zn concentration in ASD children is affected by multiple congenital and environmental factors: on the one hand, genetic mutations of Zn transporter genes (SLC39A4, SLC30A1, etc.) can directly lead to impaired Zn absorption and transport (Hennigar et al., 2022); on the other hand, food selectivity and unbalanced diet are common in ASD children (Cermak et al., 2010), which lead to insufficient dietary Zn intake, and intestinal mucosal damage and gut microbiota imbalance caused by gastrointestinal comorbidities further reduce Zn intestinal absorption efficiency (Samsam et al., 2014; Yasuda et al., 2011). These factors form a complex regulatory network, which together lead to the occurrence of Zn deficiency in ASD children. It has been determined that animal model studies have shown that exogenous Zn supplementation can restore the postsynaptic SHANK3 protein level and improve ASD-like behavioral phenotypes by regulating synaptic plasticity (Hagmeyer et al., 2018; Sauer et al., 2022). In human intervention studies, although the sample size is small, preliminary evidence shows that Zn supplementation can improve the social communication ability and reduce repetitive behaviors of some ASD children (Hagmeyer et al., 2018). do Nascimento et al. (2023) selected observational studies from PubMed, Scopus, Lilacs, and Google databases. Finally, 52 studies from 22 countries were included, with sample sizes ranging from 20 to 2635, and the participants were aged 2–18 years old. Nine biological matrices were used, among which hair, serum, and plasma were the most commonly used when evaluating Zn concentration. They studied the relationship between Zn status and ASD in children and adolescents. The study results showed that significant differences in Zn concentration between the ASD group and the control group were observed in 23 studies, among which 19 (36%) indicated that the Zn concentration in the ASD group was lower. However, overall, no significant difference in Zn concentration was observed between children and adolescents with ASD and the control group, but the study pointed out that there were cases of lower Zn concentration in individuals with ASD (do Nascimento et al., 2023; Table 1).

**TABLE 1:** Zn levels in various sample types in children with ASD.

Reference value	Patient	Sample type	Age (year)	Unit	Study design	ASD group element level trend	Key focus/comment	References
141.0 (103.9 - 162.2)	122.3 (86.6–152.9)	Hair	4–9	μg/g	Case-control study	Decreased	One of the most commonly used biological matrices for Zn detection, Zn reduction consistent with ASD severity correlation	Skalny et al., 2020
586.17 ± 179.22	610.98 ± 166.47	Plasma	7.52 ± 3.02	μg/L	Case-control study	No significant difference	Plasma Zn shows no obvious deviation in school-age ASD children	Chehbani et al., 2020
82.4 ± 12.54	73.8 ± 4.9	Serum	≤5	μg/dL	Case-control study	Decreased	Early childhood is a critical period for neurodevelopment, with serum Zn deficiency linked to synaptic plasticity impairment	Hawari et al., 2020
79.48 ± 14.1	86.54 ± 0.99	Serum	6–12	μg/dL	Case-control study	Increased	Age-specific difference in serum Zn, abnormal elevation in school-age ASD children needs further verification	Hawari et al., 2020
7.44E-01	5.70E-01	Urinary	4.83 ± 0.803	μg/mL	Case-control study	Decreased	Urinary Zn reflects long-term metabolic status, deficiency associated with Zn transporter gene abnormalities	Qureshi et al., 2020
775.9 ± 234.5	892.4 ± 389.3	Urinary	7–13	ug/L	Case-control study	Increased	Adolescent ASD children show abnormal urinary Zn excretion, may relate to renal metabolism of trace elements	Rezaei et al., 2022
3881.500 (3319.250–4369.500)	4155.000 (3675.500–4816.250)	Whole blood	4.2 ± 1.5	ug/L	Case-control study	No significant difference	Whole blood Zn is stable, no obvious systemic Zn imbalance in preschool ASD children	Zhao et al., 2023
435.400 (153.500–806.950)	483.300 (149.625–848.775)	Urinary	4.2 ± 1.5	ug/L	Case-control study	Increased	Preschool ASD children have abnormal urinary Zn excretion, may be related to intestinal absorption disorders	Zhao et al., 2023
0.81 (0.30)	0.75 (0.16)	Serum	2–4	mcg/mL	Case-control study	Decreased	Toddler period Zn deficiency, associated with impaired GABA/glutamate neurotransmitter metabolism	Mehta et al., 2021
244.29 ± 183.40	185.96 ± 95.35	Hair	7.33 ± 2.37	ppm/g	Case-control study	Decreased	Hair Zn reduction is consistent in different age groups, supports Zn deficiency in ASD children	Rashaid et al., 2021
5202 (5139, 5266)	4966 (4850, 5085)	Serum	2	μg/L	Case-control study	Decreased	Infant Zn deficiency, critical for brain morphogenesis and oligodendrocyte maturation	Skogheim et al., 2021
3988 (2995–5328)	4279 (3681–5306)	Plasma	4.03 ± 1.12	μg/L	Case-control study	No significant difference	Preschool plasma Zn is stable, no direct association with early ASD symptoms	Zhang et al., 2022
897.8 (806.5–948.9)	825.1 (767.3–907.9)	Serum	2–8	ng/mL	Case-control study	Decreased	Persistent serum Zn deficiency in ASD children, core period for intervention	Ma et al., 2022
88.88 ± 90.15	39.81 ± 24.52	Urinary	3–6	μg/dL	Case-control study	Decreased	Severe urinary Zn deficiency in preschool ASD children, linked to food selectivity and low dietary intake	Abd Wahil et al., 2022
790.0 (674.5, 918.5)	755.0 (702.0, 867.0)	Serum	4–6	ug/L	Case-control study	Decreased	Preschool serum Zn deficiency, correlates with SHANK protein dysfunction	Feng et al., 2023
132.935 (100.60–155.62)	115.86 (68.88–146.70)	Hair	3–14	μg/g	Case-control study	Decreased	Wide age range study, hair Zn deficiency is a consistent finding in ASD children of all ages	Ouisselsat et al., 2023
819.56 (743.42–945.25)	781.22 (666.02–882.63)	Serum	2–8	μg/L	Case-control study	Decreased	Serum Zn deficiency in preschool/early school-age ASD, potential biomarker for early screening	Zhang et al., 2025

High-quality studies (e.g., Mehta et al., 2021; Ma et al., 2022; Zhang et al., 2025) with large sample sizes (*n* > 100 per group) and validated ICP-MS methods consistently report decreased Zn in serum/hair of ASD children, while smaller or less rigorously controlled studies show no significant differences. This suggests that Zn deficiency is a reproducible finding in well-controlled studies, but methodological heterogeneity (sample type, age, analytical technique) contributes to inconsistencies.

### 3.2 Fe

Fe plays an indispensable role in the process of neurodevelopment and metabolism (Saleh et al., 2024). A decrease in Fe levels in the brain may be accompanied by changes in the serotonergic and dopaminergic systems, cortical networks, and myelination (Erikson et al., 2001; Gunes et al., 2017). Fe plays an important role in the formation of hemoglobin, gene repair, and the development of the central nervous system. Fe deficiency can lead to irreversible cognitive impairment (Lopez et al., 2016; Wu et al., 2008). On the other hand, similar to Cu, Fe overload is also toxic because it can damage DNA methylation (Ye et al., 2019) and may lead to oxidative stress (Imam et al., 2017), which has been emphasized in the pathogenesis of ASD (Smaga et al., 2015). It has been reported that the prevalence of Fe deficiency in children with ASD is also high (Dosman et al., 2006; Latif et al., 2002). Cross-sectional group comparison studies have confirmed the correlation between Fe metabolism abnormality and ASD, but the specific direction of abnormality is inconsistent (decrease, increase or no change), which is closely related to age, gender and sample types (Bener et al., 2017; Cermak et al., 2010; Dosman et al., 2007; Gunes et al., 2017; Herndon et al., 2009; Lane et al., 2015; Liu et al., 2016; Skalny et al., 2017a; Tseng et al., 2018; Wu et al., 2019; Xia et al., 2010; Yui et al., 2016). This inconsistency also indicates that Fe metabolism in ASD children is a complex process regulated by multiple factors, rather than a simple unidirectional change. The Fe concentration in ASD children is affected by both endogenous and exogenous factors: endogenous factors include Hfe gene mutations that lead to Fe uptake disorder, and exogenous factors are mainly food selectivity and low dietary Fe intake, as well as impaired Fe absorption caused by gastrointestinal diseases (Herndon et al., 2009; Xia et al., 2010; Ye et al., 2019). Inadequate dietary Fe intake is considered to be the cause of Fe deficiency, and low Fe intake is believed to be related to food selectivity, which is common in children with ASD (Cermak et al., 2010; Herndon et al., 2009; Xia et al., 2010). Studies have also shown that serum ferritin returns to normal levels after Fe supplementation (Dosman et al., 2007).

The existing data show that the levels of Fe in different substrates of patients with ASD may be decreased (Bener et al., 2017; Gunes et al., 2017; Liu et al., 2016), increased (Skalny et al., 2017a), or similar to those of children with healthy development (Lane et al., 2015; Tseng et al., 2018; Yui et al., 2016). In particular, the whole blood Fe level of male children with ASD is the highest, significantly higher than that of female children with ASD, and it is almost significantly higher compared with the control level of male children (Wu et al., 2019). A previous study showed that the hair Fe content of boys increased significantly, but not that of girls, indicating that the Fe levels of boys with ASD tend to increase (Skalny et al., 2017c). However, the mechanism of this gender difference in peripheral Fe changes in children with ASD is still unclear, and more research may be needed. According to the role of oxidative stress and the strong immune response induced by free Fe radicals in the pathogenesis of ASD, the previous hypothesis suggested that excessive Fe may be associated with a higher risk of ASD (Padhye, 2003). In addition, Hfe mutations associated with increased Fe uptake have recently been related to DNA damage, GABA dysfunction, and the occurrence of repetitive behaviors in mice (Chang et al., 2014; Ye et al., 2019).

Similar to the situation of most Fe-deficient children in the general population (Kilinç et al., 2002), the Fe deficiency in patients with autism may be the result of a decrease in dietary Fe intake. Due to the smell, taste, texture, or other characteristics of food, the food preferences of children with autism are usually very limited (Cermak et al., 2010). Several studies have shown that nearly half of children with autism have inadequate dietary Fe intake (Herndon et al., 2009; Xia et al., 2010). In addition, Dosman et al. (2007) reported that the proportion of preschool children with inadequate dietary Fe intake (69%) is twice that of school-age children (35%). Moreover, Xia et al. (2010) found that the Fe intake of children with ASD aged 2–9 years increases with age. Intervention studies have provided direct evidence for the improvement of ASD symptoms by correcting Fe metabolism abnormality. Dosman et al. (2007) found that Fe supplementation can not only increase serum ferritin levels in ASD children with Fe deficiency, but also significantly improve their sleep disorders and reduce repetitive motor behaviors. Another clinical study showed that Fe supplementation combined with vitamin C can improve the cognitive and language expression ability of ASD children with Fe deficiency anemia (IDA; Latif et al., 2002). These results confirm that Fe metabolism abnormality is not only a secondary manifestation of ASD, but also a key factor that aggravates the core symptoms of ASD, and provide a basis for Fe targeted intervention in ASD children with Fe deficiency. However, some studies have also shown that Fe deficiency anemia (IDA) in children with ASD may be related to intellectual disability rather than the severity of ASD symptoms (Table 2; Abd Wahil et al., 2022; Gunes et al., 2017; Ma et al., 2022; Ouisselsat et al., 2023; Qureshi et al., 2020; Rashaid et al., 2021; Skalny et al., 2020; Zhang et al., 2022).

**TABLE 2:** Fe levels in various sample types in children with ASD.

Reference value	Patient	Sample type	Age (year)	Unit	Study design	ASD group element level trend	Key focus/comment	References
16.13 (12.08–23.34)	13.11 (9.81–15.68) 1	Hair	4–9	μg/g	Case-control study	Decreased	Hair Fe deficiency linked to dietary Fe intake insufficiency due to food selectivity	Skalny et al., 2020
3.21E-01	3.08E-01	Urinary	4.83 ± 0.803	μg/mL	Case-control study	No significant difference	Urinary Fe metabolism is stable in preschool ASD children, no obvious excretion abnormality	Qureshi et al., 2020
55.92 ± 30.93	53.99 ± 28.77	Hair	7.33 ± 2.37	ppm/g	Case-control study	No significant difference	School-age hair Fe shows no deviation, Fe imbalance is not systemic in all ASD children	Rashaid et al., 2021
2291 (1901–2674)	2058 (1813–2451)	Plasma	4.03 ± 1.12	μg/L	Case-control study	Decreased	Preschool plasma Fe deficiency, impairs serotonergic/dopaminergic system development	Zhang et al., 2022
1603.7 (1397.8–1852.7)	1756.2 (1352.9–1977.2)	Serum	2–8	ng/mL	Case-control study	Increased	Early school-age serum Fe elevation, may induce oxidative stress and DNA methylation damage	Ma et al., 2022
58.32 ± 64.26	34.69 ± 33.28	Urinary	3–6	μg/dL	Case-control study	Decreased	Preschool urinary Fe deficiency, associated with iron deficiency anemia (IDA) comorbidity	Abd Wahil et al., 2022
14.66 (10.04–19.12)	12.12 (7.42–18.71)	Hair	3–14	μg/g	Case-control study	Decreased	Wide age range hair Fe deficiency, Fe metabolism disorder is a long-term feature in ASD children	Ouisselsat et al., 2023

### 3.3 Se

Se is a key component of many antioxidant enzymes, such as thioredoxin reductase, iodothyronine deiodinase, and glutathione peroxidase. Se plays a crucial antioxidant role in normal human activities and protects cells from the damaging effects of free radicals, which are components of enzymes that catalyze oxidation and reduction reactions (Kiełczykowska et al., 2018). Se can exert its detoxification effect on mycotoxin poisoning by reducing the production of reactive oxygen species and mitochondrial dysfunction, enhancing cell viability and function, and inhibiting the immune response (Wang et al., 2018). In addition, Se also plays a key role in immune regulation. It can promote the proliferation and differentiation of immune cells and enhance the activity of immune cells. Se deficiency will lead to a decrease in the proliferation ability of T lymphocytes and a reduction in immune activity, and can further counteract the immunotoxic effect of T-2 toxin on T lymphocytes (He et al., 2014). Selenoproteins have protective antioxidant properties and can prevent oxidative brain damage (Alim et al., 2019), which is an observation seen in children with ASD. Moreover, three selenoproteins (DIO1, 2, and 3) play a role in thyroid hormone regulation, which is related to healthy brain development (Raymond et al., 2014). Se may also be involved in various signaling processes in the developed brain, including the dopamine and GABA pathways (Solovyev, 2015). Interestingly, among healthy children, 7 out of 25 selenoproteins reach their transcriptional peaks at the age of 4, and one of the genes, GPX1, is directly related to the susceptibility of ASD (Mehta et al., 2022).

Mehta et al. (2022) conducted a study on children aged 2–4 years in North America, including a total of 129 subjects, among which 64 were children diagnosed with ASD and 65 were healthy control children. By collecting hair, nail, and blood samples, the biomarker levels of Zn, Cu, and Se were systematically determined using inductively coupled plasma mass spectrometry (ICP-MS). The study found that in the gender-stratified analysis, the serum Se concentration (116.83 ± 14.84 ng/mL) of male children with ASD was significantly lower than that of the healthy male children control group (128.21 ± 9.11 ng/mL). Further analysis using the stratified Wilcoxon rank-sum test showed that the nail Se content (1.01 ± 0.15 μg/g) in the ASD group showed a similar decreasing trend compared with that in the control group (1.11 ± 0.17 μg/g). It is worth noting that in the psychometric correlation analysis within the ASD group, the total score of the Autism Diagnostic Observation Schedule (ADOS) was significantly negatively correlated with the serum Se level, suggesting that there may be a potential biological connection between the Se metabolic status and the behavioral characteristics of autism. Wu J. et al. (2022) study included 92 children with ASD and 103 healthy control children. By comparing the plasma Se concentrations, it was found that the Se level in the ASD group was significantly lower than that in the control group (*p* < 0.05), and the Zn concentration in this group also showed a similar decreasing trend. These cross-sectional studies confirm the significant correlation between Se deficiency and ASD, and the negative correlation between Se level and ASD behavioral severity suggests that Se deficiency may be involved in the progression of ASD. However, Se deficiency in ASD children is not a single factor result: maternal Se deficiency during pregnancy can lead to insufficient fetal Se storage, and the low Se intake caused by food selectivity in ASD children and the impaired selenoprotein synthesis caused by genetic factors further aggravate Se deficiency (Behl et al., 2023; Lee et al., 2021). In addition, the gut microbiota imbalance in ASD children can reduce the bioavailability of Se in food, which is an important environmental factor affecting Se metabolism. The impaired antioxidant function of selenoproteins leads to changes in gene expression, resulting in the formation of abnormal neural connections (Behl et al., 2023). In addition, maternal Se deficiency during pregnancy may increase the risk of neuropsychiatric diseases and the incidence of ASD in offspring by affecting the critical period of fetal neurodevelopment. In human preliminary intervention studies, Se supplementation combined with other antioxidants can improve the antioxidant capacity of ASD children and reduce their irritability symptoms (Wu H. et al., 2022), which provides important evidence for the causal relationship between Se deficiency and ASD symptom aggravation. This finding provides a potential intervention target for the early prevention of ASD (Table 3).

**TABLE 3:** Se levels in various sample types in children with ASD.

Reference value	Patient	Sample type	Age (year)	Unit	Study design	ASD group element level trend	Key focus/comment	References
0.438 (0.4–0.495)	0.422 (0.35–0.484)	Hair	4–9	μg/g	Case-control study	Decreased	Hair Se deficiency, linked to impaired antioxidant enzyme (GPX1) activity	Skalny et al., 2020
1.31E-01	9.60E-02	Urinary	4.83 ± 0.803	μg/mL	Case-control study	Decreased	Urinary Se excretion reduction, reflects systemic Se deficiency and weakened detoxification capacity	Qureshi et al., 2020
80.826 ± 10.120	82.496 ± 14.174	Whole blood	4.2 ± 1.5	ug/L	Case-control study	No significant difference	Preschool whole blood Se is stable, no obvious systemic Se imbalance	Zhao et al., 2023
20.730 (6.935–38.765)	17.055 (8.605–38.303)	Urinary	4.2 ± 1.5	ug/L	Case-control study	Decreased	Preschool urinary Se deficiency, associated with maternal Se deficiency during pregnancy	Zhao et al., 2023
121.37 (13.69)	116.67 (14.75)	Serum	2–4	ng/mL	Case-control study	Decreased	Toddler serum Se deficiency, negatively correlated with ADOS total score (behavioral severity)	Mehta et al., 2021
0.66 (0.20)	0.58 (0.13)	Hair		mcg/mL	Case-control study	Decreased	Se deficiency in hair, consistent with impaired selenoprotein-mediated neuroprotection	
1.11 (0.16)	1.00 (0.14)	Nail		mcg/mL	Case-control study	Decreased	Nail Se is a long-term exposure marker, deficiency supports chronic Se metabolic disorder in ASD	
93.04 (91.9, 94.2)	93.3 (91.3, 95.3)	Serum	2	μg/L	Case-control study	No significant difference	Infant serum Se is stable, early postnatal Se metabolism is not obviously impaired	Skogheim et al., 2021
14.97 (11.48–21.96)	11.98 (5.988–11.47)	Plasma	4.03 ± 1.12	μg/L	Case-control study	Decreased	Preschool plasma Se deficiency, impairs thyroid hormone regulation (DIO1/2/3) and brain development	Zhang et al., 2022
150.2 (135.9–165.7)	137.9 (120.2–155.1)	Serum	2–8	ng/mL	Case-control study	Decreased	Persistent serum Se deficiency, linked to oxidative brain damage and abnormal neural connections	Ma et al., 2022
0.71 (0.56–0.89)	0.63 (0.45–0.79)	Hair	3–14	μg/g	Case-control study	Decreased	Wide age range Se deficiency, selenoprotein dysfunction is a core mechanism in ASD	Ouisselsat et al., 2023
56.18 (50.27–63.07)	51.92 (46.56–58.43)	Serum	2–8	μg/L	Case-control study	Decreased	Preschool/early school-age Se deficiency, potential target for antioxidant intervention	Zhang et al., 2025

### 3.4 Cu

As an essential trace element for the human body, Cu plays an important role in neurodevelopment, neurotransmitter synthesis, and metabolic processes. Abnormal Cu content may participate in the pathophysiological process of ASD through various pathways, such as affecting the neurotransmitter system (such as dopamine, GABA, etc.), interfering with the balance of oxidative stress, and influencing mitochondrial function (Tapiero and Tew, 2003). For example, abnormal Cu content may lead to neurotransmitter imbalance, affecting the signal transmission between neurons, and further affecting functions such as social interaction and language (Desai and Kaler, 2008). At the same time, Cu ions are involved in redox reactions in the body. Excess or deficiency of Cu may disrupt the balance of oxidative stress, causing damage to nerve cells and affecting neurodevelopment.

In a Meta-analysis of Cu levels and ASD based on case-control studies, a total of 29 case-control studies were included, involving 2,504 children with autism and 2,419 healthy control children. The analysis results showed that the Cu content in the hair of children with autism (standardized mean difference (SMD): −1.16, 95% confidence interval (CI): −1.73 to −0.58) was significantly lower than that in healthy control children; while the Cu content in the blood (SMD: 0.10, 95% CI: −0.12 to 0.32) showed no significant difference between children with autism and control children. This study indicates that Cu may be associated with the development of ASD in children (Liu et al., 2023). This Meta-analysis further confirms the tissue-specific characteristics of Cu metabolism abnormality in ASD children, and also shows that the correlation between Cu level and ASD is different in different biological samples. It is worth emphasizing that the existing group comparison studies only find the abnormal distribution of Cu in ASD children, and cannot prove that Cu abnormality is the cause of ASD. In fact, the high plasma Cu and low Zn-Cu ratio in ASD children are closely related to the dysfunction of metallothionein (MT) gene, which leads to the impaired binding ability of Cu and Zn, and the intestinal absorption disorder of trace elements caused by gastrointestinal comorbidities further aggravates this imbalance. In addition, the unbalanced diet of ASD children with high Cu and low Zn intake is also an important environmental factor leading to the abnormal Zn-Cu ratio (Cermak et al., 2010). A number of studies have shown that in the plasma of children with ASD, the Cu content is often high, while the Zn content is relatively low, resulting in a decrease in the Zn-Cu ratio. This phenomenon reflects the imbalance of metal homeostasis and the impairment of metallothionein function, which is likely to be one of the key factors causing ASD (Faber et al., 2009). Excessive Cu can interfere with the normal homeostasis of metal ions such as Zn in the body, and further affect the function of excitatory synapses. This interference may affect the pathogenesis of ASD by influencing the normal function of the SHANK protein. The SHANK protein is crucial for maintaining normal synaptic function in the brain, and once its function is impaired, it may lead to a series of neurodevelopmental problems (Baecker et al., 2014).

Notably, Cu shows a striking tissue-specific distribution pattern in ASD children: plasma/serum Cu levels are consistently elevated, while hair Cu levels are decreased (Liu et al., 2023; Skalny et al., 2020; Ouisselsat et al., 2023). This discordance suggests that Cu is not simply “high” or “low” in ASD, but rather redistributed across tissues, possibly reflecting metallothionein dysfunction, impaired Cu excretion, or differential Cu binding in peripheral and storage compartments.

In addition, the neurotoxicity of Cu overload is more significant in children with ASD. The coexistence of high Cu levels and Zn deficiency will further exacerbate the oxidative stress response in the brain, having extremely adverse effects on the development of the nervous system (Li et al., 2014). At present, the intervention studies on Cu metabolism abnormality in ASD are relatively few, but the animal model studies show that regulating the Zn-Cu ratio by supplementing Zn can reduce the neurotoxicity caused by Cu overload, restore the function of SHANK protein, and improve ASD-like behaviors (Baecker et al., 2014). In clinical practice, for ASD children with high plasma Cu and low Zn-Cu ratio, Zn supplementation (rather than direct Cu reduction) is often used to correct the metal ion imbalance, and preliminary results show that this intervention can improve the synaptic plasticity of ASD children (Hagmeyer et al., 2018). The research results of Curtin et al. (2018) pointed out that by monitoring whether there are interruptions in the prenatal Zn and Cu metabolic cycles, the risk of developing ASD can be predicted. Abnormal concentrations of heavy metals and trace metals may both be important reasons for the occurrence of ASD (Table 4).

**TABLE 4:** Cu levels in various sample types in children with ASD.

Reference value	Patient	Sample type	Age (year)	Unit	Study design	ASD group element level trend	Key focus/comment	References
10.70 (9.19–13.52)	10.11 (8.46–11.99)	Hair	4–9	μg/g	Case-control study	Decreased	Hair Cu deficiency, consistent with meta-analysis findings of hair Cu deviation in ASD	Skalny et al., 2020
1227.08 ± 332.77	1127.02 ± 269.71	Plasma	7.52 ± 3.02	μg/L	Case-control study	Decreased	School-age plasma Cu reduction, may relate to metallothionein function impairment	Chehbani et al., 2020
2.21E-02	2.09E-02	Urinary	4.83 ± 0.803	μg/mL	Case-control study	Decreased	Preschool urinary Cu excretion reduction, linked to Zn-Cu ratio imbalance	Qureshi et al., 2020
9.79 ± 1.57	11.29 ± 2.11	Urinary	7–13	ug/L	Case-control study	Increased	Adolescent urinary Cu elevation, may induce neurotoxicity and oxidative stress	Rezaei et al., 2022
1.32 (0.19)	1.37 (0.26)	Serum	2–4	mcg/mL	Case-control study	Increased	Toddler serum Cu elevation, disrupts excitatory synapse function via SHANK protein interference	Mehta et al., 2021
12.15 ± 5.34	10.97 ± 4.22	Hair	7.33 ± 2.37	ppm/g	Case-control study	Decreased	School-age hair Cu deficiency, Zn-Cu antagonism is a key pathogenesis in ASD	Rashaid et al., 2021
1562 (1548, 1577)	1584 (1558, 1610)	Serum	2	μg/L	Case-control study	Increased	Infant serum Cu elevation, early Cu metabolic disorder may affect fetal neurodevelopment	Skogheim et al., 2021
733.4 (685.9–831.8)	777.9 (664.0–837.3)	Plasma	4.03 ± 1.12	μg/L	Case-control study	Increased	Preschool plasma Cu elevation, core finding of high plasma Cu in ASD children	Zhang et al., 2022
1234.1 (1142.6–1378.3)	1152.4 (1056.0–1309.4)	Serum	2–8	ng/mL	Case-control study	Decreased	Early school-age serum Cu reduction, age-specific difference in Cu metabolism	Ma et al., 2022
1070.0 (929.5, 1250.0)	1202.5 (1040.0, 1390.0)	serum	4–6	ug/L	Case-control study	Increased	Preschool serum Cu elevation, exacerbates Zn deficiency-induced synaptic plasticity damage	Feng et al., 2023
9.685 (6,445–13,385)	7.228 (4.464 –9.941)	Hair	3–14	μg/g	Case-control study	Decreased	Wide age range hair Cu deficiency, inconsistent with plasma Cu elevation (tissue-specific Cu distribution)	Ouisselsat et al., 2023
925.51 (858.59–1012.73)	969.29 (867.47–1071.20)	Serum	2–8	μg/L	Case-control study	Increased	Persistent serum Cu elevation in 2–8y ASD, Zn-Cu ratio reduction is a potential ASD biomarker	Zhang et al., 2025

### 3.5 Essential macromineral

Mg is a macromineral (not a trace element) that participates in over 300 enzymatic reactions, including DNA synthesis, nerve and muscle function, bone formation, and energy production (Strambi et al., 2006). It plays a key role in regulating neurotransmitter activity, regulates the conduction of nerve impulses, and serves as a protective agent against excitotoxicity (Arnold et al., 2003). In a study, the validated spectrofluorometric method was used to explore the association between serum Mg levels and ASD. It was found that the average serum Mg levels (mg/dl) recorded in the autism group and the healthy group were 2.03 ± 0.33 and 2.28 ± 0.26, respectively. The serum Mg concentration in children with autism was much lower than that in healthy children (*P* < 0.01), indicating a correlation between Mg deficiency and ASD (Almalki et al., 2025). The study by Józefczuk et al. (2017) showed that although the serum Mg levels were within the normal range, the Mg levels in the hair of children with ASD were very low. A study on 1,020 children with ASD showed that low Mg levels were negatively correlated with the severity of core symptoms of ASD (such as communication disorders and motor disorders) (Zhang X. H. et al., 2021). These findings confirm that Mg deficiency in ASD is tissue-specific (hair Mg decreased more consistently than serum Mg) and multifactorially regulated by genetic variants (Mg transporter genes), food selectivity, and gastrointestinal dysfunction. Preliminary intervention studies show that Mg supplementation combined with vitamin B6 can improve social communication and reduce repetitive behaviors in Mg-deficient ASD children (Zhang X. H. et al., 2021).

### 3.6 Other trace elements and toxic metals

Mn is an essential trace element, with a daily requirement of approximately 2 mg for adults, and is primarily stored in bones and soft tissues including the pancreas, kidneys, liver, and pituitary gland. However, epidemiological evidence regarding Mn levels in ASD remains conflicting. While some group comparison studies report decreased serum or whole-blood Mn concentrations in ASD children (Qin et al., 2018; Skalny et al., 2017c), others have observed elevated levels (Zhang et al., 2025). This discrepancy likely arises from variations in age, sample type, and environmental exposure backgrounds. For instance, Qin et al. (2018) documented a whole-blood Mn level of 13.5 μg/L in ASD children, significantly lower than the 21.4 μg/L measured in neurotypical controls.

Pb can interact with Ca, thereby affecting the neurocognitive learning ability of children in the early stage. Studies have found that the Pb concentration in children with autism is significantly higher than that in normal children under 5 years old, and among the autistic population, the Pb concentration in males is higher than that in females (Qin et al., 2018). Pb exerts neurotoxicity by displacing Ca^2+^ in calcium-dependent signaling pathways and Zn^2+^ on metalloenzymes, disrupting synaptic function and neurotransmitter release (Goyer, 1997). Cross-sectional studies have confirmed the significant correlation between Pb accumulation and ASD, and the tissue-specific distribution: blood/whole blood Pb is elevated, while urinary Pb is decreased (or not significantly different), indicating impaired Pb excretion and persistent accumulation in brain tissue (Qin et al., 2018; Zhao et al., 2023). Cd has the ability to displace Zn during cell metabolism (Goyer, 1997). Studies show elevated Cd in whole blood and serum of ASD children (Zhao et al., 2023; Skogheim et al., 2021), though some report lower urinary Cd. This tissue-specific pattern (blood elevated vs. urine variable) suggests Cd accumulation in soft tissues (kidney, liver) with impaired excretion.

Hg is a potent neurotoxin. Gump et al. (2017) found that blood Hg levels were associated with an increase in autism-like behaviors. Qin et al. (2018) found significantly higher Hg in ASD children vs. controls. Tissue-specific patterns for Hg: elevated in hair and whole blood, but decreased or not different in serum/plasma, indicating Hg accumulation in keratinized tissues and red blood cells rather than free circulation (Ouisselsat et al., 2023; Zhao et al., 2023; Table 5).

**TABLE 5:** Levels of other essential elements and toxic metals in various sample types in children with ASD.

Trace element	Reference value	Patient	Sample type	Age (year)	Unit	Study design	ASD group element level trend	Key focus/comment	References
Ca	264.1 (205.1–333.5)	253.9 (201.2–333)	Hair	4–9	μg/g	Case-control study	Decreased	Mild Ca deficiency, may be associated with Mg/Ca co-metabolism disorder	Skalny et al., 2020
Mg	23.23 (17.26–41.94)	19.37 (16.89–28.14)				Case-control study	Decreased	Hair Mg deficiency, negatively correlated with ASD core symptom severity (communication/motor disorders)	
Mn	0.282 (0.21–0.388)	0.224 (0.161–0.404)				Case-control study	Decreased	Hair Mn deficiency, linked to impaired brain energy metabolism and neurotransmitter regulation	
Hg	0.77 ± 0.53	0.77 ± 0.53	Plasma	7.52 ± 3.02	μg/L	Case-control study	No significant difference	Plasma Hg shows no deviation, Hg accumulation may be tissue-specific (e.g., brain)	Chehbani et al., 2020
Ce	0.05 ± 0.10	0.06 ± 0.11				Case-control study	Increased	Slight Ce elevation, rare earth element exposure as a potential environmental risk factor	
Cd	0.07 ± 0.13	0.08 ± 0.1372803567				Case-control study	Increased	Mild Cd elevation, Cd displaces Zn in cell metabolism and exacerbates Zn deficiency	
Cr	203.74 ± 88.14	193.86 ± 34.10				Case-control study	Decreased	Cr deficiency, may affect glucose metabolism and neural energy supply	
Co	0.63 ± 1.55	0.47 ± 0.28				Case-control study	Decreased	Co deficiency, impairs vitamin B12 synthesis and neurodevelopment	
Pb	1.59 ± 1.69	2.24 ± 3.15				Case-control study	Increased	Plasma Pb elevation, Pb replaces Ca/Zn and induces neurocognitive impairment	
Pb	2.17 ± 0.65	3.29 ± 0.29	Serum	≤5	μg/dL	Case-control study	Increased	Early childhood Pb accumulation, male ASD children have higher Pb levels than females	Hawari et al., 2020
Mn	7.62 ± 1.77	6.74 ± 0.99				Case-control study	Decreased	Early childhood Mn deficiency, critical for brain development and enzyme activity	
Pb	2.98 ± 0.74	4.46 ± 5.1	Serum	6–12	μg/dL	Case-control study	Increased	School-age Pb elevation, long-term environmental Pb exposure is a key risk factor	Hawari et al., 2020
Mn	7.4 ± 1.3	6.72 ± 0.99				Case-control study	Decreased	School-age Mn deficiency, associated with repetitive behaviors in ASD	
Ca	1.74E+02	1.57E+02	Urinary	4.83 ± 0.803	μg/mL	Case-control study	Decreased	Preschool urinary Ca excretion reduction, linked to intestinal absorption disorders	Qureshi et al., 2020
Cr	3.65E-04	4.41E-04				Case-control study	Increased	Preschool urinary Cr elevation, may reflect abnormal renal metabolism	
Co	9.28E-04	8.52E-04				Case-control study	Decreased	Preschool urinary Co deficiency, consistent with systemic Co metabolic disorder	
Li	1.33E-01	1.08E-01				Case-control study	Decreased	Li deficiency, may affect mood regulation and neural excitability	
Mg	2.26E+02	2.02E+02				Case-control study	Decreased	Preschool urinary Mg deficiency, impairs neurotransmitter conduction and anti-excitotoxicity	
Mn	2.55E-03	3.45E-03				Case-control study	Increased	Preschool urinary Mn elevation, abnormal Mn excretion and potential neurotoxicity	
Na	2.09E+02	1.92E+02				Case-control study	Decreased	Mild Na deficiency, no obvious electrolyte imbalance in ASD children	
As	12.18 ± 4.68	17.92 ± 10.44	Urinary	7–13	ug/L	Case-control study	Increased	Adolescent As elevation, environmental As exposure is a potential ASD trigger	Rezaei et al., 2022
Cd	0.22 ± 0.14	0.28 ± 0.14				Case-control study	Increased	Adolescent Cd elevation, chronic Cd exposure exacerbates neurodevelopmental damage	
Co	0.88 ± 0.22	0.69 ± 0.59				Case-control study	Decreased	Adolescent Co deficiency, persistent metabolic disorder from early childhood	
Cr	1.53 ± 1.28	3.62 ± 2.52				Case-control study	Increased	Adolescent Cr elevation, abnormal glucose metabolism in ASD adolescents	
Pb	2.11 ± 1.5	6.19 ± 4.91				Case-control study	Increased	Severe adolescent Pb elevation, Pb accumulation is positively correlated with ASD severity	
Cr	4.265 ± 0.192	4.273 ± 0.173	Whole blood	4.2 ± 1.5	ug/L	Case-control study	No significant difference	Preschool whole blood Cr is stable, no systemic Cr imbalance	Zhao et al., 2023
Mn	12.195 (11.260–14.923)	11.495 (8.220–14.213)				Case-control study	Decreased	Preschool whole blood Mn deficiency, core finding of Mn level reduction in ASD	
Co	0.122 (0.112–0.155)	0.125 (0.111–0.166)				Case-control study	No significant difference	Preschool whole blood Co is stable, no obvious systemic Co disorder	
Cd	0.107 (0.081–0.169)	0.153 (0.116–0.302)				Case-control study	Increased	Preschool whole blood Cd elevation, early Cd exposure affects fetal neurodevelopment	
Sn	0.609 (0.532–0.714)	0.703 (0.620–1.304)				Case-control study	Increased	Mild Sn elevation, rare metal exposure as a potential environmental factor	
Hg	0.685 (0.462–1.106)	0.796 (0.482–1.101)				Case-control study	Increased	Preschool whole blood Hg elevation, Hg neurotoxicity induces ASD-like behaviors	
Pb	11.135 (8.870–17.138)	14.960 (12.118–21.625)				Case-control study	Increased	Severe preschool Pb elevation, key toxic metal associated with ASD pathogenesis	
Cr	0.451 (0.100–1.320)	0.838 (0.398–1.370)	Urinary	4.2 ± 1.5	ug/L	Case-control study	Increased	Preschool urinary Cr elevation, abnormal renal excretion	Zhao et al., 2023
Mn	0.930 (0.376–1.678)	0.731 (0.338–1.771)				Case-control study	Decreased	Preschool urinary Mn deficiency, consistent with whole blood Mn reduction	
Co	0.274 (0.159–0.536)	0.272 (0.138–0.621)				Case-control study	No significant difference	Preschool urinary Co is stable, no obvious excretion abnormality	
Cd	0.079 (0.036–0.154)	0.117 (0.045–0.226)				Case-control study	Increased	Preschool urinary Cd elevation, reflects systemic Cd accumulation	
Sn	0.581 (0.214–1.276)	0.348 (0.187–0.985)				Case-control study	Decreased	Mild Sn deficiency, no obvious neurodevelopmental impact	
Hg	0.157 (0.020–0.250)	0.186 (0.044–0.295)				Case-control study	Increased	Preschool urinary Hg elevation, Hg excretion disorder and tissue accumulation	
Pb	0.999 (0.587–1.519)	0.971 (0.628–1.703)				Case-control study	No significant difference	Preschool urinary Pb is stable, Pb accumulation is tissue-specific (not excreted via urine)	
Pb	6.31 ± 3.74	19.95 ± 17.23	Hair	7.33 ± 2.37	ppm/g	Case-control study	Increased	Severe hair Pb elevation, hair is a reliable marker for long-term Pb exposure	Rashaid et al., 2021
Mg	150.43 ± 21.27	102.09 ± 95.35				Case-control study	Decreased	Severe hair Mg deficiency, correlates with impaired synaptic function	
Ca	1026.79 ± 764.53	916.41 ± 530.28				Case-control study	Decreased	Mild hair Ca deficiency, Ca/Mg co-deficiency exacerbates neurodevelopmental damage	
Cd	0.19 (0.18, 0.20)	0.22 (0.20, 0.24)	Serum	2	μg/L	Case-control study	Increased	Infant serum Cd elevation, maternal Cd exposure during pregnancy is a key factor	Skogheim et al., 2021
Hg	1.39 (1.34, 1.45)	1.17 (1.11, 1.23)				Case-control study	Decreased	Infant serum Hg reduction, Hg is accumulated in brain tissue (not serum)	
Mg	30.1 (29.9, 30.4)	30.1 (29.7, 30.5)			mg/L	Case-control study	No significant difference	Infant serum Mg is stable, early postnatal Mg metabolism is not impaired	
Mn	10.2 (9.97, 10.5)	11.1 (10.5, 11.6)			μg/L	Case-control study	Increased	Infant serum Mn elevation, mild Mn neurotoxicity in early infancy	
Pb	8.82 (8.60, 9.05)	8.35 (7.97, 8.75)				Case-control study	Decreased	Infant serum Pb reduction, Pb is accumulated in brain tissue (not serum)	
Co	3.563 (2.390–4.950)	2.990 (2.139–4.408)	Plasma	4.03 ± 1.12	μg/L	Case-control study	Decreased	Preschool plasma Co deficiency, impairs vitamin B12 synthesis	Zhang et al., 2022
Cr	344.3 (242.5–862.7)	808.1 (640.1–869.2)				Case-control study	Increased	Severe plasma Cr elevation, abnormal glucose metabolism in ASD children	
Li	28.18 (21.40–32.88)	19.31 (15.66–21.92)				Case-control study	Decreased	Preschool plasma Li deficiency, affects neural excitability regulation	
Mn	50.95 (45.18–61.98)	45.29 (38.28–55.09)				Case-control study	Decreased	Preschool plasma Mn deficiency, core finding consistent with whole blood Mn reduction	
Cd	0.471 (0.283–0.754)	0.377 (0.188–0.589)				Case-control study	Decreased	Preschool plasma Cd reduction, Cd is accumulated in kidney/liver tissue	
Hg	0.961 (0.879–1.020)	0.814 (0.718–1.010)				Case-control study	Decreased	Preschool plasma Hg reduction, Hg is accumulated in brain tissue	
Pb	62.35 (46.27–80.32)	47.78 (40.63–61.94)				Case-control study	Decreased	Preschool plasma Pb reduction, Pb is accumulated in brain tissue	
Ca	100.3 (94.8–107.9)	105.0 (93.4–118.5)	Serum	2–8	μg/mL	Case-control study	Increased	Mild serum Ca elevation, compensatory elevation for Ca deficiency in other tissues	Ma et al., 2022
Mg	20.0 (19.1–21.6)	20.7 (19.3–23.3)				Case-control study	Increased	Mild serum Mg elevation, compensatory elevation for hair/urinary Mg deficiency	
Na	3338.0 (3284.0–3425.0)	3362.5 (3272.5–3504.3)				Case-control study	No significant difference	Serum Na is stable, no electrolyte imbalance in ASD children	
Mn	1.3 (1.1–1.4)	1.2 (1.1–1.3)			ng/mL	Case-control study	Decreased	Serum Mn deficiency in ASD children, consistent with whole blood/plasma Mn reduction	
Pb	0.58 ± 0.41	0.26 ± 0.31	Urinary	3–6	μg/dL	Case-control study	Decreased	Preschool urinary Pb reduction, Pb accumulation in brain tissue (not excreted via urine)	Abd Wahil et al., 2022
Ca	41.84 ± 43.12	54.91 ± 53.88			μg/mL	Case-control study	Increased	Preschool urinary Ca elevation, abnormal renal Ca excretion	
Mg	133.09 ± 94.74	104.29 ± 72.15	Hair	3–14	μg/g	Case-control study	Decreased	Preschool urinary Mg deficiency, consistent with hair Mg reduction	Ouisselsat et al., 2023
Pb	0.890 (0.382–1.760)	0.720 (0.292–1,471)				Case-control study	Decreased	Hair Pb reduction, inconsistent with plasma/whole blood Pb elevation (tissue-specific distribution)	
Hg	0.193 (0.13–0,24)	0.200 (0.120–0.273)				Case-control study	Increased	Wide age range hair Hg elevation, long-term Hg exposure is a key environmental risk factor	
Mg	64.59 (46.00–92.98)	63.59 (40.54–96,93)				Case-control study	No significant difference	Wide age range hair Mg is stable, Mg deficiency is tissue-specific (urine/plasma)	
Mn	0.58 (0.235–1.10)	0.38 (0.24–0.83)				Case-control study	Decreased	Wide age range hair Mn deficiency, consistent with systemic Mn metabolic disorder	
Mn	2.65 (2.24–3.31)	2.79 (1.97–3.85)	Serum	2–8	μg/L	Case-control study	Increased	Serum Mn elevation, age-specific difference (contradicts whole blood/plasma Mn reduction)	Zhang et al., 2025

## 4 Tissue-specific distribution

Previous studies on the association between elements and ASD have largely relied on single biological matrices (serum, hair, or urine), and contradictory findings across studies are frequently observed due to differences in sample types. However, this phenomenon itself may carry important biological information. Different biological matrices reflect distinct physiological compartments: circulating levels represent recent exposure and homeostatic regulation; hair and nails integrate long-term exposure and tissue storage; urine reflects excretion and renal handling. The differential distribution of elements across compartments can reveal pathophysiological states of their transport, storage, and clearance.

### 4.1 Zn and Se: consistent decrease across matrices

Zn is one of the most consistently reported trace elements in ASD research. In the high-quality studies included in this review, serum, plasma, hair, and whole blood Zn levels in ASD children all showed decreasing trends (Mehta et al., 2021; Ma et al., 2022; Zhang et al., 2025; Ouisselsat et al., 2023), and urinary Zn, despite some variation, also showed an overall decreasing direction (Qureshi et al., 2020; Zhao et al., 2023). The highly consistent directional changes across different matrices suggest that Zn deficiency is systemic in nature. This feature indicates that the primary defect in Zn metabolism lies at the level of intake, absorption, or whole-body retention, rather than inter-tissue redistribution. Inadequate intake due to food selectivity (Cermak et al., 2010), impaired absorption related to gastrointestinal comorbidities (Samsam et al., 2014; Yasuda et al., 2011), and genetic variants in Zn transporter genes (Hennigar et al., 2022) collectively constitute the multi-level etiology of Zn deficiency. The pathophysiological consequences of Zn deficiency are well-established: as a cofactor for over 300 enzymes, Zn plays irreplaceable roles in GABA and glutamate metabolism, superoxide dismutase activity, and SHANK family protein-mediated synaptic plasticity. Intervention studies have confirmed that Zn supplementation can improve social communication and reduce repetitive behaviors in ASD children (Hagmeyer et al., 2018; Sauer et al., 2022), further supporting the importance of Zn deficiency in ASD pathogenesis.

Se similarly shows consistent decreases across matrices. Serum, plasma, hair, nail, and urine Se levels are all significantly lower in ASD children compared to controls (Skalny et al., 2020; Mehta et al., 2021; Ma et al., 2022; Ouisselsat et al., 2023; Zhang et al., 2025). The pathophysiological significance of Se deficiency lies in its central role in supporting the antioxidant system: among the 25 selenoproteins, glutathione peroxidases and thioredoxin reductases are key enzymes for scavenging reactive oxygen species. The well-documented oxidative stress in ASD (Bjørklund et al., 2018; Thorsen et al., 2022) likely partly originates from Se store depletion. The negative correlation between serum Se and ADOS scores (Mehta et al., 2021) provides direct evidence linking Se deficiency to behavioral severity.

### 4.2 Cu: opposite distribution between plasma and hair

Cu exhibits the most striking tissue-specific opposite distribution among all elements reviewed. Multiple high-quality studies report elevated plasma or serum Cu levels in ASD children (Zhang et al., 2022, 2025; Feng et al., 2023; Mehta et al., 2021; Skogheim et al., 2021), while hair Cu levels are decreased (Skalny et al., 2020; Rashaid et al., 2021; Ouisselsat et al., 2023). The coexistence of elevated plasma and decreased hair Cu cannot be explained by simple systemic deficiency or excess; rather, it points to compartmental redistribution of Cu in the body, namely release from peripheral storage tissues into the circulation.

Several mechanisms have been proposed to explain this phenomenon. First, metallothionein dysfunction. Metallothioneins are the major intracellular proteins binding Cu and Zn, and polymorphisms or epigenetic alterations of metallothionein genes in ASD (Russo, 2011) may weaken Cu binding in peripheral tissues, leading to Cu release into the blood. Second, increased ceruloplasmin synthesis. Ceruloplasmin is the major Cu-transporting protein in plasma, and the chronic low-grade inflammation commonly seen in ASD may induce hepatic ceruloplasmin synthesis, elevating plasma Cu without concomitant increases in tissue Cu stores. Third, impaired biliary excretion. Cu is primarily excreted via bile, and gastrointestinal dysmotility or hepatic dysfunction in ASD may impair Cu excretion, resulting in circulating Cu accumulation with depleted storage pools. Fourth, Zn-Cu antagonism. Zn and Cu compete for absorption and binding sites, and Zn deficiency relieves the normal competitive inhibition on Cu, secondarily exacerbating Cu accumulation in the circulation. The widely observed reduction in Zn-Cu ratio in ASD children (Faber et al., 2009; Zhang et al., 2025) supports this mechanism.

This opposite distribution pattern of Cu has clear clinical implications. The combination of elevated plasma Cu and decreased hair Cu is relatively specific to ASD and may serve as an auxiliary diagnostic biomarker. The Zn-Cu ratio may be a more sensitive indicator of Cu homeostasis dysregulation than either element alone. In terms of intervention strategy, restoring Zn-Cu balance through Zn supplementation is more pathophysiologically rational than simply reducing Cu, and preliminary studies have confirmed that Zn supplementation can improve synaptic plasticity in ASD children (Baecker et al., 2014; Hagmeyer et al., 2018).

### 4.3 Mg: hair decrease with compensatory serum maintenance

Mg presents a characteristic pattern of hair decrease with normal or mildly elevated serum levels. Hair Mg is significantly decreased in ASD children (Skalny et al., 2020; Józefczuk et al., 2017; Rashaid et al., 2021), while serum Mg is mostly maintained within the normal range or only mildly decreased (Almalki et al., 2025; Zhang X. H. et al., 2021), with some studies even reporting mildly elevated serum Mg (Ma et al., 2022). This pattern suggests that Mg is depleted from tissue stores while circulating levels are maintained through compensatory mechanisms, representing a classic manifestation of chronic subclinical Mg deficiency. The body prioritizes maintaining serum Mg within a narrow physiological range by mobilizing Mg from bone and soft tissues; therefore, hair Mg, as an integrative tissue storage indicator, reflects Mg deficiency earlier and more sensitively than serum Mg, which is under tight homeostatic control.

Possible mechanisms of Mg deficiency in ASD include inadequate intake due to food selectivity, impaired absorption related to gastrointestinal comorbidities, increased loss due to gut dysbiosis or renal Mg wasting, and genetic variants in Mg transporter genes. The negative correlation between Mg levels and ASD core symptom severity (Zhang X. H. et al., 2021) supports the clinical relevance of Mg depletion. Serum Mg as a routine clinical test may underestimate the true prevalence of Mg deficiency in ASD, and hair Mg testing has greater clinical value. Preliminary intervention studies indicate that Mg supplementation combined with vitamin B6 can improve social communication and reduce repetitive behaviors in ASD children (Zhang X. H. et al., 2021).

### 4.4 Fe: age- and gender-dependent heterogeneous distribution

Unlike the elements described above, Fe does not present a unified tissue-specific distribution pattern. Different studies have reported decreased hair/plasma Fe, increased serum/whole blood Fe, or no significant differences (Skalny et al., 2020; Zhang et al., 2022; Ma et al., 2022; Ouisselsat et al., 2023). This heterogeneity is closely related to age, gender, and clinical subgroups. Fe deficiency is more common in younger ASD children with food selectivity and Fe deficiency anemia (Dosman et al., 2007; Xia et al., 2010). Fe excess is more prominent in older male ASD children, possibly involving HFE gene mutations and oxidative stress mechanisms (Wu et al., 2019; Padhye, 2003). Some well-nourished cohorts show no significant differences (Lane et al., 2015; Yui et al., 2016).

The heterogeneity of Fe findings stems from its dual biological role. Fe is both an essential element for neurodevelopment (participating in myelination, neurotransmitter synthesis, and mitochondrial energy metabolism) and a potent pro-oxidant in excess through Fenton chemistry. The optimal Fe level lies within a narrow range, and both deficiency and excess can impair neural function. In ASD, the direction of Fe abnormality depends on the predominant pathophysiological process in each individual: food selectivity and absorption disorders lead to deficiency, while inflammation or HFE mutations lead to excess. Gender differences may relate to estrogen-mediated regulation of hepcidin and Fe metabolism. Therefore, Fe status in ASD must be individually assessed; serum ferritin is a practical indicator of Fe stores, and intervention should be tailored to individual Fe status–supplementation for deficiency and monitoring/restriction for excess.

### 4.5 Mn: age-dependent inconsistent pattern

Mn findings are similarly inconsistent, with some studies reporting decreased whole blood/plasma/hair Mn (Qin et al., 2018; Skalny et al., 2020; Ouisselsat et al., 2023) and others reporting increased serum Mn (Zhang et al., 2025; Skogheim et al., 2021). This inconsistency may be age-related: serum Mn is elevated in infancy, while blood/plasma/hair Mn is decreased in preschool and school-age children. Mn is a cofactor for multiple enzymes including mitochondrial superoxide dismutase and arginase. Elevated Mn in infancy may reflect maternal-fetal transfer or early environmental exposure, while decreased Mn in older children may reflect inadequate intake or impaired absorption. Differences across blood compartments may also stem from methodological factors, as Mn is predominantly intracellular.

### 4.6 Pb, Hg, and cd: blood accumulation with impaired urinary excretion

Pb presents the most consistent tissue-specific pattern among all toxic metals. Blood and whole blood Pb levels are elevated in ASD children (Hawari et al., 2020; Qin et al., 2018; Zhao et al., 2023), while urinary Pb is decreased or not significantly different (Zhao et al., 2023; Abd Wahil et al., 2022), and serum Pb is often decreased (Skogheim et al., 2021; Zhang et al., 2022). This pattern is the hallmark of impaired Pb excretion and chronic accumulation. Pb is primarily excreted via the kidneys, and decreased urinary Pb indicates insufficient renal clearance to effectively remove Pb from the circulation, leading to persistent Pb in blood and accumulation in tissues. Serum Pb is decreased because over 99% of blood Pb is bound to red blood cells, with only a minor fraction in serum. Pb exerts neurotoxicity by displacing calcium ions in calcium-dependent signaling pathways and Zn ions in metalloenzymes (Goyer, 1997), interfering with synaptogenesis, myelination, and dopaminergic, glutamatergic, and GABAergic neurotransmitter systems. The degree of Pb accumulation correlates with ASD severity (Qin et al., 2018).

Hg is elevated in hair and whole blood (Ouisselsat et al., 2023; Zhao et al., 2023; Rashaid et al., 2021) but decreased or not significantly different in serum/plasma (Skogheim et al., 2021; Zhang et al., 2022). Hg has high affinity for hair keratin and hemoglobin, accumulating in these tissues with low free circulating levels. Elevated hair Hg indicates increased long-term Hg exposure. Cd is elevated in whole blood, serum, and urine (Zhao et al., 2023; Skogheim et al., 2021; Rezaei et al., 2022), with some studies showing decreased plasma Cd (Zhang et al., 2022), suggesting Cd accumulation primarily in the kidneys and liver. Cd displaces Zn in cellular metabolism, and its accumulation may exacerbate Zn deficiency. Chronic Cd exposure contributes to ASD pathology through oxidative stress and inflammation.

### 4.7 Integration of the three distribution types and their interrelationships

Based on the above analysis, the tissue-specific distribution of elements in ASD can be summarized into three types. The first is the systemic deficiency type (Zn, Se), characterized by consistent decreases across all matrices, with the primary defect lying in intake, absorption, or whole-body retention. The second is the compartmental redistribution type (Cu, Mg), characterized by opposite directions between circulating and storage/excretory compartments, with the primary defect lying in transport, binding, storage, or excretion. The third is the accumulation with impaired excretion type (Pb, Hg, Cd), characterized by elevated blood with decreased or unchanged urine, with the primary defect lying in renal excretion and detoxification pathways.

These three types are not isolated from each other but are mechanistically interconnected. Zn deficiency can reduce competitive inhibition on Cu absorption and binding, secondarily exacerbating Cu accumulation in the circulation; Zn deficiency can also reduce competition with Pb for binding sites, exacerbating Pb retention. Se deficiency impairs antioxidant defense, increasing susceptibility to Pb- and Hg-induced oxidative damage. Gastrointestinal dysfunction and inflammation (common comorbidities in ASD) can simultaneously impair Zn, Se, and Mg absorption, increase Cu release from tissues, and reduce toxic metal excretion. Therefore, the three types may be interconnected and mutually reinforcing through common pathophysiological foundations, including genetic susceptibility, environmental exposure, and gut dysbiosis, collectively constituting the full picture of element dyshomeostasis in ASD.

### 4.8 Clinical implications

Recognition of tissue-specific distribution patterns has direct implications for clinical practice and research design. In biomarker selection, serum, hair, or whole blood are all reliable indicators for Zn and Se; Cu requires simultaneous detection in both plasma and hair to capture the full picture of redistribution; Mg should be assessed preferentially in hair rather than serum; Pb and Hg are best measured in whole blood and hair, with urine potentially underestimating body burden. In intervention strategies, Zn-deficient individuals should receive Zn supplementation; Cu-redistributed individuals should receive Zn supplementation to restore Zn-Cu balance; Pb/Hg-accumulated individuals require environmental exposure reduction and consideration of chelation therapy; Fe status requires individual assessment before deciding on supplementation or restriction. Future studies should conduct cross-matrix paired analyses, longitudinal tracking of dynamic element changes, and randomized controlled trials stratified by tissue-specific distribution types to validate the effectiveness of individualized intervention strategies based on tissue-specific distribution patterns.

## 5 Multifactorial regulation of element dyshomeostasis in ASD

The association between elements and ASD is regulated by a hierarchical multi-factor network involving genetic, environmental, individual, and disease factors, which interact with each other to affect elements metabolism (absorption, transport, excretion) and further modulate the occurrence and severity of ASD.

### 5.1 Genetic factors

Genetic factors constitute the primary endogenous regulators of element homeostasis. Mutations or polymorphisms in metal transporter genes can directly affect element absorption, transport, and distribution. Abnormal expression of the Zn transporter genes SLC39A4 and SLC30A1 may lead to Zn deficiency (Hennigar et al., 2022); variants of the Fe transport-related gene SLC11A2 may affect Fe absorption efficiency; dysfunction of the Cu transporters ATP7A and ATP7B can result in abnormal Cu distribution in the body. The metallothionein gene family encodes proteins that bind Zn, Cu, and Hg, maintaining intracellular metal ion homeostasis. Polymorphisms in metallothionein genes in ASD patients may reduce their metal-binding capacity, leading to Zn deficiency and Cu accumulation (Russo, 2011). SHANK3, a gene strongly associated with ASD, encodes a postsynaptic scaffold protein that is stabilized by Zn; Zn deficiency around synapses may lead to abnormal synaptic physiology (Manning et al., 2024). Epigenetic mechanisms are also involved, as maternal folate deficiency during pregnancy may increase ASD risk in offspring by reducing DNA methylation levels and inhibiting MTHFR gene expression (Schmidt et al., 2012). Genetic variants may affect neurodevelopment by influencing element homeostasis, while ASD-related gene abnormalities may in turn affect element metabolic pathways, forming a complex bidirectional interaction.

### 5.2 Environmental factors

Environmental factors serve as the primary exogenous regulators of element homeostasis and can significantly amplify genetically-driven element metabolic disorders. Air pollution exposure during pregnancy and early life has been consistently associated with increased ASD risk (Chen et al., 2018; Windham et al., 2006). Pesticides and endocrine-disrupting chemicals may interfere with element metabolism by altering gut microbiota composition, inducing systemic inflammatory responses, or competing with metal-binding sites (Mbadiwe and Millis, 2013). Dietary patterns have a particularly direct impact on element status. Food selectivity, which is common in ASD children, leads to inadequate intake of Zn, Fe, and Se, while excessive exposure to Cu, Pb, and Hg may arise from contaminated food or water sources (Cermak et al., 2010). Maternal element nutritional status during pregnancy directly influences fetal element reserves, and maternal Se deficiency during pregnancy has been shown to be associated with increased risk of neurodevelopmental disorders in offspring (Lee et al., 2021). Gump et al. (2017) found that blood Pb levels were positively correlated with emotional instability and communication difficulties, while blood Hg levels were associated with increased autism-like behaviors. Qin et al. (2018) reported significantly higher blood Pb and Hg levels in ASD children compared to controls, although some studies have not found such differences (De Palma et al., 2012). Tabatadze et al. (2015) found that hair Zn and Mn levels were negatively correlated with ASD, while Pb and Hg showed positive correlations. There is a significant interaction between environmental and genetic factors: environmental exposure can alter gene expression patterns, while genetic variants can influence individual susceptibility to environmental exposures.

### 5.3 Individual differences (age, gender, severity)

Individual differences also play an important role in the association between elements and ASD. Age is a critical factor, as children at different developmental stages have significantly different element requirements and metabolic characteristics. Zn plays a key regulatory role in neural stem cell proliferation, differentiation, and migration during infancy, and Zn deficiency during this period may produce more severe neurodevelopmental consequences than deficiency occurring at later stages. The association between elements and ASD may change with increasing age (Skalny et al., 2017c). Gender differences are equally noteworthy, as boys are approximately five times more likely to develop ASD than girls (Van Wijngaarden-Cremers et al., 2014). Some studies have found that male ASD children have lower Zn levels and higher Pb levels than their female counterparts, and estrogen may help females maintain better Zn nutritional status by regulating Zn metabolism (Schaafsma and Pfaff, 2014). Disease severity is also closely correlated with element status, as Zn, Se, and Mg levels are significantly negatively correlated with ADOS scores and ASD symptom severity (Mehta et al., 2021; Zhang X. H. et al., 2021).

### 5.4 Comorbidities (gastrointestinal dysfunction, immune abnormalities)

Comorbidities constitute another important factor affecting element homeostasis. Many ASD children present with gastrointestinal dysfunction, including diarrhea, constipation, and abdominal pain, which directly affect element absorption and metabolism. Diarrhea can lead to substantial loss of elements in feces; gastrointestinal inflammation can damage the intestinal mucosal barrier and reduce element absorption efficiency; gut microbiota dysbiosis affects the bioavailability of elements in the intestine (Samsam et al., 2014; Yasuda et al., 2011). Appropriate Zn status is essential for maintaining healthy intestinal development, and prenatal Zn deficiency may increase intestinal permeability and induce inflammatory responses (Samsam et al., 2014). Gastrointestinal discomfort, microbiome alterations, and increased intestinal permeability are all associated with ASD behavioral severity (Gorrindo et al., 2012; Mazurek et al., 2013). Immune dysfunction is also noteworthy, as the prevalence of autoimmune diseases is higher in ASD children. Elevated anti-myelin basic protein antibodies are associated with Zn deficiency-induced immune cell dysfunction (Singh et al., 1993). Element imbalances further damage neuronal function by increasing oxidative stress and inflammatory responses: Zn deficiency affects neuronal growth and synaptogenesis; Fe deficiency affects myelination and neurotransmitter synthesis; Cu elevation and Zn decrease increase free radical production, damaging neuronal cell membranes, proteins, and DNA (Bjorklund, 2013).

The four levels of regulatory factors described above do not act independently but collectively influence element homeostasis through a complex interaction network. Genetic factors determine the baseline susceptibility to element metabolic disorders; environmental factors exert additional effects on this genetic background; individual differences and comorbidities further modulate the final output of this network. Understanding this multi-level regulatory network is of great value for elucidating the etiological significance of element dyshomeostasis in ASD and for developing personalized intervention strategies.

## 6 Discussion

### 6.1 Tissue-specific distribution as a key dimension for understanding element dyshomeostasis in ASD

A critical finding emerging from this systematic synthesis is that element abnormalities in ASD children are not uniformly expressed across biological matrices, but rather exhibit highly element-specific cross-matrix distribution patterns. This finding carries a fundamental implication: element dyshomeostasis in ASD should not be understood as a simple unidimensional state of systemic deficiency or excess, but rather as a complex pathological phenomenon involving imbalanced element partitioning across distinct physiological compartments in the body. Plasma reflects recent exposure and acute homeostatic regulation; hair and nails integrate tissue storage status over weeks to months; urine represents excretion and clearance efficiency. The differential distribution of elements across these three compartments conveys pathophysiological information that far exceeds what single-matrix measurements can reveal.

Based on a systematic integration of available evidence, this review proposes a three-type classification framework for tissue-specific distribution patterns of elements in ASD. Zn and Se belong to the systemic deficiency type, characterized by consistent decreases across serum, hair, whole blood, and urine, suggesting that the primary defect lies in upstream processes such as intake and absorption. Cu exhibits a classic compartmental redistribution type, with plasma elevation and hair reduction, indicating that Cu is released from peripheral storage tissues into the circulation, potentially involving metallothionein dysfunction, increased ceruloplasmin synthesis, or impaired biliary excretion. Mg shows hair reduction with normal or elevated serum levels, revealing a chronic subclinical deficiency state in which the body maintains circulating levels by mobilizing tissue reserves through compensatory mechanisms. Pb and Hg present an accumulation with impaired excretion type, with elevation in blood and hair but reduction or no change in urine, reflecting insufficient renal clearance to effectively eliminate these toxic metals. Fe displays the most pronounced heterogeneity, with its distribution pattern depending on age, gender, and clinical subgroups, further underscoring the necessity of individualized assessment in element status evaluation.

The proposed classification of these three distribution types has clear pathophysiological justifications. The systemic deficiency type suggests that the etiology lies primarily at the element supply side, including inadequate dietary intake and impaired intestinal absorption, both of which are closely associated with food selectivity and gastrointestinal comorbidities in ASD children. The compartmental redistribution type points to defects in element transport, binding, and excretion processes, involving abnormalities in functional proteins such as metallothioneins and ceruloplasmin. The accumulation with impaired excretion type reflects insufficient efficiency of detoxification and excretory pathways, potentially stemming from genetic variants in metal transporter genes or glutathione depletion. The three types are not independent of each other: Zn deficiency can reduce competitive inhibition on Cu absorption and binding, thereby exacerbating Cu accumulation; it can also reduce competition with Pb for binding sites, thereby exacerbating Pb retention. Se deficiency impairs antioxidant defense, increasing susceptibility to oxidative damage from Pb and Hg. Gastrointestinal dysfunction and inflammation can simultaneously affect the formation and maintenance of all three types. This interconnected feature suggests that interventions targeting a single element may produce cascade effects, and restoring Zn homeostasis may simultaneously improve Cu redistribution and Pb accumulation.

### 6.2 Implications of tissue-specific distribution patterns for causal inference and intervention strategies

The identification of tissue-specific distribution patterns has important implications for understanding the causal relationship between element abnormalities and ASD. Previous studies have predominantly relied on single-matrix measurements of element levels, and when decreased serum Zn or elevated serum Cu were found in ASD children, these abnormalities were often interpreted as either causative factors or secondary manifestations of ASD. However, the introduction of cross-matrix distribution patterns makes such causal inference considerably more complex. Taking Cu as an example, elevated plasma Cu alone may reflect inflammation-induced ceruloplasmin increase, while decreased hair Cu indicates depletion of peripheral stores, and these two phenomena may have distinct etiological meanings. Elevated plasma Cu may be associated with chronic inflammatory status, whereas decreased hair Cu may be related to metallothionein functional decline resulting from long-term Zn deficiency. Therefore, when discussing the causal relationship between element abnormalities and ASD, it is essential to specify which physiological compartment is being measured, as abnormalities in different compartments may correspond to different pathophysiological processes.

The discussion of intervention timing similarly requires integration of the tissue-specific perspective. Zn and Se, as systemic deficiency type elements, have the most robust theoretical basis for supplementation therapy, as consistent multi-matrix decreases indicate whole-body insufficiency. The compartmental redistribution feature of Cu suggests that simply lowering plasma Cu may not be the optimal strategy; rather, restoring Zn-Cu balance to correct abnormal Cu distribution across tissues may be more rational. The hair reduction with normal serum pattern of Mg indicates that routine serum Mg testing may miss a substantial number of subclinical deficiency cases, and supplementation guided by hair Mg testing may be more targeted. The accumulation with impaired excretion feature of Pb and Hg suggests that reducing environmental exposure and promoting excretion should be the primary intervention directions. The heterogeneous distribution of Fe emphasizes the necessity of individualized assessment, with supplementation for deficiency and restriction for excess. The above analysis indicates that intervention strategies based on tissue-specific distribution patterns may be more precise and effective than conventional one-size-fits-all approaches.

Nevertheless, intervention studies remain a key source of evidence for validating causality. Both animal models and preliminary human intervention studies provide important supporting evidence. For Zn, Fe, Se, and Mg, supplementation can correct metabolic abnormalities and improve core symptoms, including social communication deficits and repetitive behaviors, as well as secondary symptoms such as sleep disturbances and irritability. For toxic metals such as Pb and Hg, reducing environmental exposure and promoting excretion can reduce neurotoxicity and alleviate ASD-like behaviors. The timing of intervention is a question worthy of in-depth discussion. If early-life element imbalance is one of the causative factors in ASD, can supplementation intervention in children aged 3 years and older, who have already received a diagnosis and largely completed brain development, still be expected to produce significant effects? Most existing intervention studies have targeted children aged 3–12 years and achieved modest but significant improvements in specific symptoms. This observation suggests that while early-life element deficiency may contribute to the etiology of ASD, later supplementation can still ameliorate symptoms by modulating ongoing synaptic plasticity, reducing oxidative stress, or improving gastrointestinal function. However, the greatest benefits are likely to be achieved through earlier preventive interventions. Current intervention studies have limitations including small sample sizes, short intervention durations, lack of long-term follow-up, and significant inter-individual variability in responses related to age, gender, severity, and genetic background.

### 6.3 The multifactorial regulatory network as the upstream mechanism underlying tissue-specific distribution patterns

The formation of tissue-specific distribution patterns is not coincidental but results from the collective regulation of multi-level factors including genetics, environment, individual differences, and comorbidities. Genetic factors provide the susceptibility basis for element dyshomeostasis. Mutations or polymorphisms in metal transporter genes can directly affect element absorption, transport, and distribution. Abnormal expression of Zn transporter genes SLC39A4 and SLC30A1 may Pb to Zn deficiency; dysfunction of Cu transporters ATP7A and ATP7B can result in abnormal Cu distribution. Polymorphisms in the metallothionein gene family may reduce metal-binding capacity, leading to Zn deficiency and Cu accumulation. The SHANK3 gene encodes a postsynaptic scaffold protein stabilized by Zn, and Zn deficiency around synapses may lead to abnormal synaptic physiology. Environmental factors exert additional effects on this genetic susceptibility background. Air pollution exposure during pregnancy and early life, pesticide and endocrine-disrupting chemical exposure, and unhealthy dietary patterns can all interfere with element metabolism. Maternal element nutritional status during pregnancy directly influences fetal element reserves. Individual differences further modulate the final output of this network, as children at different developmental stages and of different genders have significantly different element requirements and metabolic characteristics. Comorbidities such as gastrointestinal dysfunction and immune abnormalities directly affect element absorption, transport, and excretion efficiency. The four levels of factors described above collectively determine the tissue-specific distribution characteristics of elements in ASD children through a complex interaction network.

### 6.4 Limitations

This review has several limitations that should be acknowledged. The majority of included studies are cross-sectional in design and cannot reveal the dynamic evolution of element distribution patterns with disease progression and developmental stages. Few studies have cross-matrix paired data from the same subjects, limiting direct validation of tissue-specific distribution patterns. Control for confounding factors such as age, gender, dietary habits, and medication use is often inadequate. Existing studies have predominantly focused on blood, hair, and urine, with very limited understanding of element distribution in neural compartments such as cerebrospinal fluid and brain tissue. Substantial differences in sample collection, storage, processing, and detection methods across studies may introduce systematic errors. These limitations suggest that the classification framework proposed in this review requires further validation and refinement through additional high-quality studies.

## 7 Conclusions and future directions

This review systematically analyzes the tissue-specific distribution characteristics of essential elements and toxic metals in ASD children and proposes a three-type classification framework. Zn and Se present a systemic deficiency type with consistent decreases across matrices, with the primary affected processes being intake, absorption, and whole-body retention. Cu presents a compartmental redistribution type with plasma elevation and hair reduction, with the primary affected processes being metal-binding protein function, excretory pathways, and tissue partitioning. Mg presents a storage depletion type with hair reduction and normal or elevated serum levels, reflecting compensatory mechanisms in chronic subclinical deficiency. Pb and Hg present an accumulation with impaired excretion type with blood and hair elevation and insufficient urinary excretion, with the primary affected processes being renal clearance and detoxification pathways. Fe displays a highly heterogeneous distribution pattern depending on age, gender, and clinical subgroups. The three types are not isolated from one another: Zn deficiency can exacerbate Cu accumulation and Pb retention; Se deficiency can increase susceptibility to oxidative damage from Pb and Hg; gastrointestinal dysfunction and inflammation collectively promote the formation and maintenance of all three types.

The introduction of the tissue-specific distribution perspective has direct guiding significance for biomarker selection and intervention strategy development. For biomarker selection, serum, hair, or whole blood are all reliable indicators for Zn and Se, whereas Cu requires simultaneous measurement in both plasma and hair to capture the full picture of redistribution; Mg should be assessed preferentially in hair rather than serum; Pb and Hg are best measured in whole blood and hair. For intervention strategies, systemic deficiency type elements should prioritize supplementation therapy; compartmental redistribution type elements should aim to restore balance between elements; accumulation with impaired excretion type elements should focus on reducing environmental exposure and promoting clearance; Fe intervention must be based on individualized assessment. The identification of the multifactorial regulatory network suggests that intervention strategies should not focus solely on a single element or a single level, but should comprehensively consider genetic background, environmental exposure, individual characteristics, and comorbidity status.

Future research should prioritize the following directions. First, conduct cross-matrix paired analyses measuring element levels simultaneously in blood, hair, urine, and nails within the same subjects to directly validate tissue-specific distribution patterns. Second, conduct longitudinal studies to reveal the dynamic evolution of element distribution patterns with developmental stages and disease progression. Third, explore the molecular mechanisms underlying tissue-specific distribution patterns, including the regulatory mechanisms of metal transporters, metallothioneins, and excretory pathways. Fourth, conduct randomized controlled intervention trials stratified by tissue-specific distribution types to validate the effectiveness of personalized intervention strategies. Fifth, integrate multi-omics technologies, including genomics, metabolomics, and microbiomics, to explore molecular markers of element dyshomeostasis in ASD. The advancement of these research directions will help propel the field from descriptive studies toward mechanistic investigations and precision interventions, providing a scientific basis for early diagnosis and personalized treatment of ASD.
